# Stealth and Biocompatible Gold Nanoparticles through Surface Coating with a Zwitterionic Derivative of Glutathione

**DOI:** 10.1021/acs.langmuir.4c01123

**Published:** 2024-05-29

**Authors:** Vinicius S. Guido, Paulo H. Olivieri, Milena L. Brito, Benedito C. Prezoto, Diego S. T. Martinez, Maria Luiza V. Oliva, Alioscka A. Sousa

**Affiliations:** † Department of Biochemistry, Federal University of São Paulo, São Paulo 04044-020, Brazil; ‡ Brazilian Nanotechnology National Laboratory (LNNano), Brazilian Center for Research in Energy and Materials (CNPEM), Campinas, São Paulo 13083-100, Brazil; § Laboratory of Pharmacology, the Butantan Institute, São Paulo 05503-900, Brazil

## Abstract

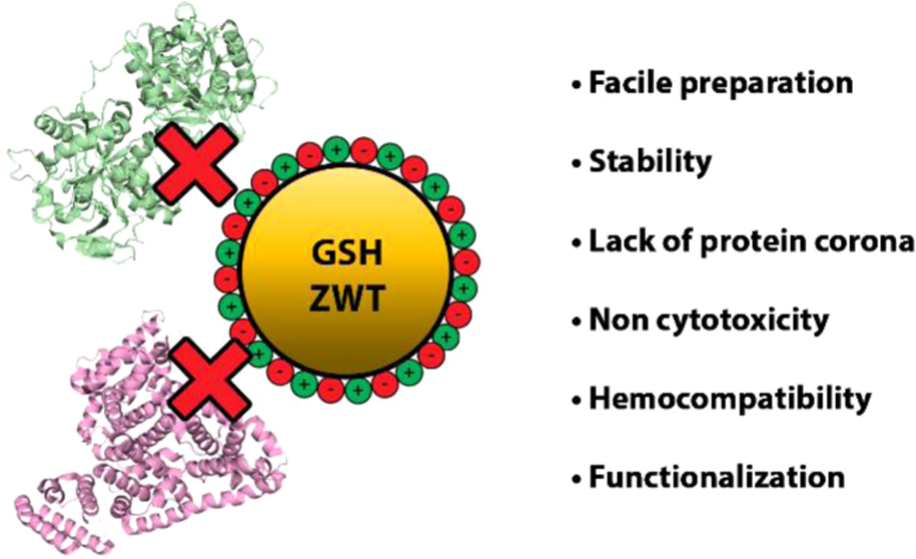

Gold nanoparticles (AuNPs) hold promise in biomedicine, but challenges like aggregation, protein corona formation, and insufficient biocompatibility must be thoroughly addressed before advancing their clinical applications. Designing AuNPs with specific protein corona compositions is challenging, and strategies for corona elimination, such as coating with polyethylene glycol (PEG), have limitations. In this study, we introduce a commercially available zwitterionic derivative of glutathione, glutathione monoethyl ester (GSH_zwt_), for the surface coating of colloidal AuNPs. Particles coated with GSH_zwt_ were investigated alongside four other AuNPs coated with various ligands, including citrate ions, tiopronin, glutathione, cysteine, and PEG. We then undertook a head-to-head comparison of these AuNPs to assess their behavior in biological fluid. GSH_zwt_-coated AuNPs exhibited exceptional resistance to aggregation and protein adsorption. The particles could also be readily functionalized with biotin and interact with streptavidin receptors in human plasma. Additionally, they exhibited significant blood compatibility and noncytotoxicity. In conclusion, GSH_zwt_ provides a practical and easy method for the surface passivation of AuNPs, creating “stealth” particles for potential clinical applications.

## Introduction

Among the myriad of inorganic nanoparticles (NPs) developed to date, gold nanoparticles (AuNPs) have emerged as the most extensively investigated due to their exceptional optical, electronic, and physicochemical properties, coupled with their ease of synthesis and bioconjugation capabilities.^1−4^ However, despite the promising potential of AuNPs in biomedical applications, there remain significant challenges that must be addressed for their widespread clinical adoption. Two primary obstacles that AuNPs face on their path to successful clinical translation are aggregation and unintended interactions with proteins.^5−7^ In particular, when AuNPs are immersed in serum or plasma, they become immediately covered with a stable layer of adsorbed biomolecules formed mainly of proteins. This “biomolecular corona” endows AuNPs with a new biological identity and influences their bioresponses.^8−11^

Despite over a decade of research on the biomolecular corona, designing AuNPs that can adopt specific corona compositions and architectures has proven to be a significant challenge.^12,13^ Consequently, a major strategy in nanomedicine has been to engineer NPs that are resistant to biomolecular adsorption, thereby eliminating the biomolecular corona altogether.^14−16^ Modification of the particle surface with hydrophilic polymers, especially polyethylene glycol (PEG), is a widely used strategy to prevent nonspecific adsorption and endow NPs with “stealth” characteristics. However, the ability of PEG to prevent protein adsorption hinges on several critical factors, including the length, architecture, and grafting density of the PEG chains covering the AuNP surface.^17,18^ In addition, PEG minimizes protein adsorption but does not completely avoid it, and repeated PEG exposure in vivo induces the production of PEG-specific antibodies.^19,20^ PEGylation can also substantially increase the overall hydrodynamic diameter (HD) of AuNPs. To overcome the limitations associated with PEG conjugation, various other protective coatings can be utilized, including peptides, proteins, lipids, polymers, and zwitterionic molecules, each with its own set of distinct advantages and disadvantages.^15,21−27^ Zwitterionic molecules, in particular, have been increasingly pursued as superior nonfouling coatings for inorganic NPs.^28−30^ Zwitterionic coatings are surrounded by a tightly bound, electrostatically induced hydration layer. This layer creates a large energy barrier and a strong repulsive force against nonspecific protein adsorption.^31−33^ However, many zwitterionic ligands developed to date entail complicated synthesis and characterization procedures, which can pose challenges for laboratories lacking expertise in organic synthesis.^15,34−40^ The lack of facile protocols to prepare zwitterionic-coated NPs may ultimately hinder further development and testing of these nanomaterials, thereby delaying their real-world clinical applications.

In this study, we demonstrate the successful use of a commercial, readily available, and biocompatible zwitterionic derivative of glutathione (GSH), glutathione monoethyl ester (GSH_zwt_), in the preparation of 5 nm colloidal AuNPs. Inorganic NPs within this size range have recently garnered increased attention for cancer nanomedicine because of their unique physiological behaviors and biological functions, including efficient renal clearance, deeper tumor penetration, and more uniform tumor distribution.^41−44^ Herein, we show that these novel GSH_zwt_-coated AuNPs exhibit high resistance against both aggregation and protein adsorption in biological media. Moreover, through a series of biocompatibility tests, we establish their significant blood compatibility and noncytotoxic properties.

## Results and Discussion

**Figure 1:**
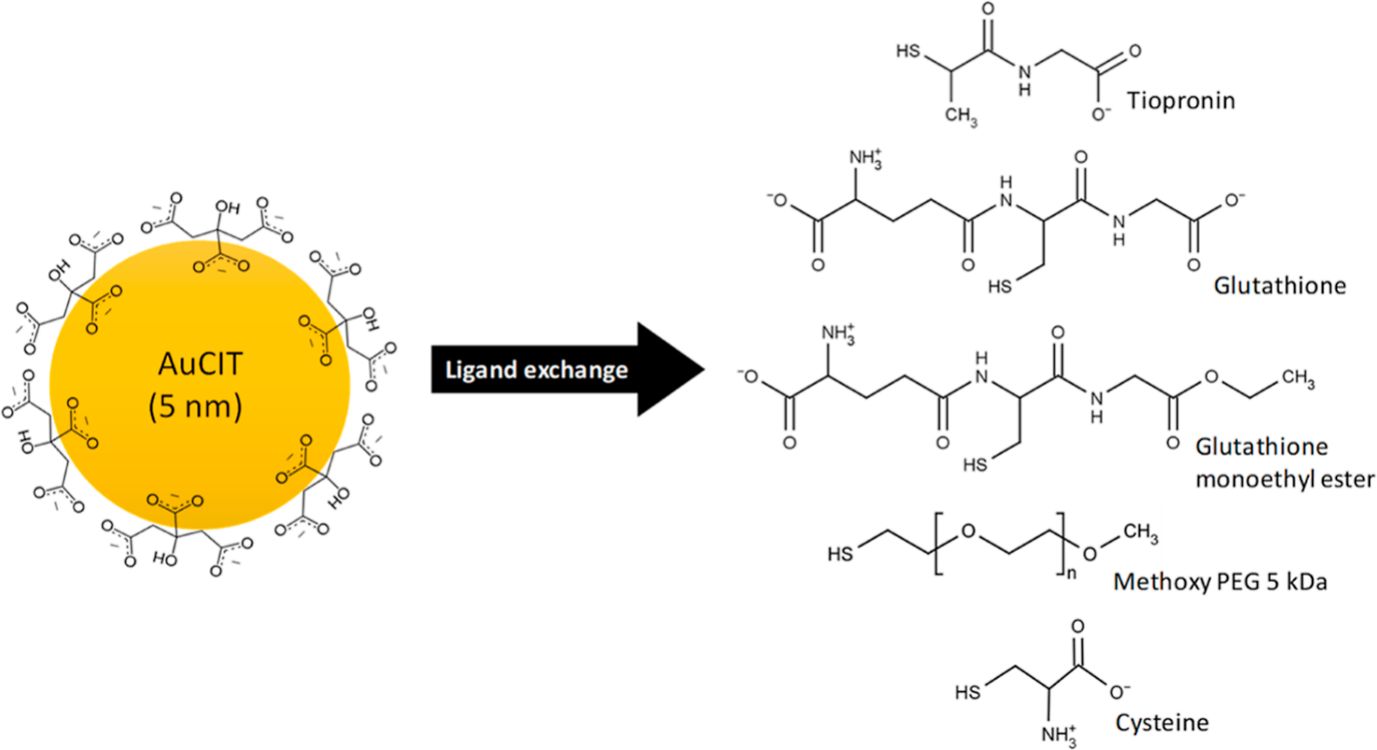
Preparation of ligand-coated AuNPs by ligand exchange of citrate-stabilized colloidal AuNPs.

We prepared a series of ligand-coated AuNPs by immobilization of each ligand onto citrate-stabilized colloidal AuNPs 5 nm in size. The selected ligands were tiopronin (TPN), GSH, GSH_zwt_, the amino acid cysteine (Cys), and methoxy PEG (Figure 1). TPN has a single charge of −1, whereas GSH contains a zwitterionic group on one end and a lone carboxylate group on the other end of the molecule, resulting in a net charge of −1. GSH_zwt_ shares a similar structure to GSH but lacks the lone carboxylate group, which confers on GSH_zwt_ a true zwitterionic character. Similarly to GSH_zwt_, Cys also possesses a zwitterionic group. The PEG ligand had a molecular weight of 5 kDa and was neutrally charged. Hereafter, we refer to the citrate-stabilized AuNPs as AuCIT, while the TPN-, GSH-, GSH_zwt_-, Cys-, and PEG-coated AuNPs are denoted as AuTPN, AuGSH, AuGSH_zwt_, AuCys, and AuPEG, respectively.

We undertook a head-to-head comparison of the different AuNPs with respect to their resistance to aggregation and protein adsorption, as well as their blood compatibility in vitro. In our comparative studies, AuCIT and AuTPN represented anionic colloidal AuNPs that were expected to exhibit strong interactions with proteins. Furthermore, these AuNPs were anticipated to show poor blood compatibility in the in vitro tests. Conversely, the behavior of AuGSH in biological environments was uncertain, as the GSH ligand possesses a net charge of −1 but also incorporates a zwitterionic group. The presence of the zwitterionic moiety suggested the potential for AuGSH to resist protein adsorption and to demonstrate blood compatibility in vitro. It is important to note that GSH is commonly used in the preparation of highly stable and biocompatible ultrasmall AuNPs and Au nanoclusters (*d* < 2nm), with numerous successful in vivo studies demonstrated to date.^45,46^ Owing to its zwitterionic surface ligand, AuGSH_zwt_ was hypothesized to resist aggregation and protein adsorption, in addition to being blood compatible. In previous studies by our research group, GSH_zwt_ has proven to be a suitable choice for creating ultrasmall AuNPs with colloidal stability in biological environments and high resistance against protein interactions.^47−49^ Regarding AuCys, it was expected to share similar properties to AuGSH_zwt_ due to the zwitterionic character of Cys. Lastly, AuPEG was anticipated to exhibit strong resistance against protein adsorption and high blood compatibility in vitro, making it a suitable benchmark for comparison with AuGSH_zwt_.

### Characterization of AuNPs

The AuNPs were characterized in phosphate buffer solution (10 mM, pH 7.4) without NaCl, using a combination of ultraviolet–visible (UV–vis) spectroscopy, dynamic light scattering (DLS), and zeta potential (ZP) measurements. The UV–vis analysis revealed a red shift of the localized surface plasmon resonance (LSPR) band for all ligand-coated AuNPs compared to AuCIT (Figure S1). DLS measurements indicated a small increase in the HD for AuTPN, AuGSH, and AuGSH_zwt_ relative to AuCIT, while AuPEG exhibited a more substantial HD increase due to the larger size of the PEG coating (Table 1, column under “buffer”). AuCys unexpectedly displayed a large HD, which was likely attributed to NP aggregation. Previous studies have observed that Cys used as a surface ligand can result in colloidally unstable NPs.^50,51^ ZP measurements revealed negative surface charges for AuTPN, AuGSH, AuPEG, and AuCys, while AuGSH_zwt_ displayed ZP values close to zero, as expected (Table 1, column under “buffer”). The ZP of AuPEG was markedly negative, consistent with observations reported in other studies.^52,53^

**Table 1:** Characterization of Protein Corona Formation on AuNPs through DLS and ZP Measurements^a^

AuNP	buffer		BSA corona		transferrin corona		FBS corona	
	diameter (nm)	ZP (mV)	diameter (nm)	ZP (mV)	diameter (nm)	ZP (mV)	diameter (nm)	ZP (mV)
AuCIT	5.1 ± 0.9	–33.9 ± 5.8	20.9 ± 3.5	–22.9 ± 2.3	16.5 ± 2.6	–25.9 ± 4.7	16.4 ± 5.7	–24.1 ± 4.5
AuTPN	6.4 ± 1.2	–23.9 ± 4.9	20.4 ± 5.2	–28.4 ± 3.8	14.4 ± 1.2	–32.2 ± 2.3	24.0 ± 3.8	–26.8 ± 2.4
AuGSH	6.4 ± 1.2	–20.8 ± 5.6	21.3 ± 2.6	–25.4 ± 2.7	11.8 ± 0.8	–21.5 ± 9.5	22.5 ± 5.2	–27.3 ± 3.8
AuGSH_zwt_	6.6 ± 0.9	0.4 ± 2.5	6.7 ± 0.6	2.6 ± 3.3	6.3 ± 0.9	–1.9 ± 1.3	6.4 ± 2.1	–0.70 ± 4.1
AuPEG	22.6 ± 4.7	–24.2 ± 3.0	23.0 ± 4.5	–23.2 ± 2.3	23.9 ± 7.1	–24.1 ± 2.3	23.8 ± 3.0	–23.5 ± 2.5
AuCys	20.7 ± 2.2	–8.8 ± 1.6	33.8 ± 4.6	–19.0 ± 5.9	38.8 ± 6.8	–18.7 ± 5.0	34.8 ± 3.4	–17.8 ± 4.7

a AuNPs were incubated in solutions containing BSA (10 mg/mL), transferrin (2 mg/mL), or fetal bovine serum (FBS) (30%) for 1 h at 37 °C. Subsequently, the AuNPs were centrifuged and washed 3×, followed by redispersion in phosphate buffer. AuNPs dispersed in buffer alone were used as a control.

### Colloidal Stability

To assess the colloidal stability of the AuNPs under physiologically relevant conditions, the particles were dispersed in phosphate buffer solution containing 150 mM NaCl and then characterized by UV–vis spectroscopy. We found that only AuGSH_zwt_ and AuPEG remained colloidally stable over a period of 24 h (not shown). To further investigate their stability, similar experiments were performed by dispersing the AuNPs in cell culture medium, which contains not only Na^+^ but also divalent cations (especially Ca^2+^ and Mg^2+^) and various small molecules. We observed that AuCIT, AuTPN, AuGSH, and AuCys aggregated rapidly, whereas AuGSH_zwt_ and AuPEG remained stable over 24 h (Figure S2). The high stability of AuGSH_zwt_ in high ionic strength solutions is attributable to a few factors: (i) the charge neutrality of the NPs, (ii) the negligible van der Waals attraction between NPs because of their relatively small size, and (iii) the presence of a tightly bound hydration layer whose removal is energetically unfavorable.^54,55^ We also examined the stability of the AuNPs across a pH range of 4 to 9. We found that AuGSH, AuGSH_zwt_, and AuPEG retained colloidal stability under the test conditions, while the other AuNPs exhibited various degrees of aggregation based on the pH and incubation time (Figure S3).

### Formation of BSA and Transferrin Coronas

We studied the formation of hard protein coronas composed of purified bovine serum albumin (BSA) and human transferrin around the surface of the AuNPs. First, the AuNPs were incubated with BSA or transferrin for 1 and 24 h. Subsequently, the particles were separated from excess and loosely bound proteins using centrifugation and washing steps, followed by redispersion of the pellets in phosphate buffer solution. The obtained AuNPs were characterized by UV–vis spectroscopy, DLS, and ZP measurements. This combination of techniques is well-established for the robust characterization of the hard protein corona surrounding colloidal AuNPs.^56^

**Figure 2:**
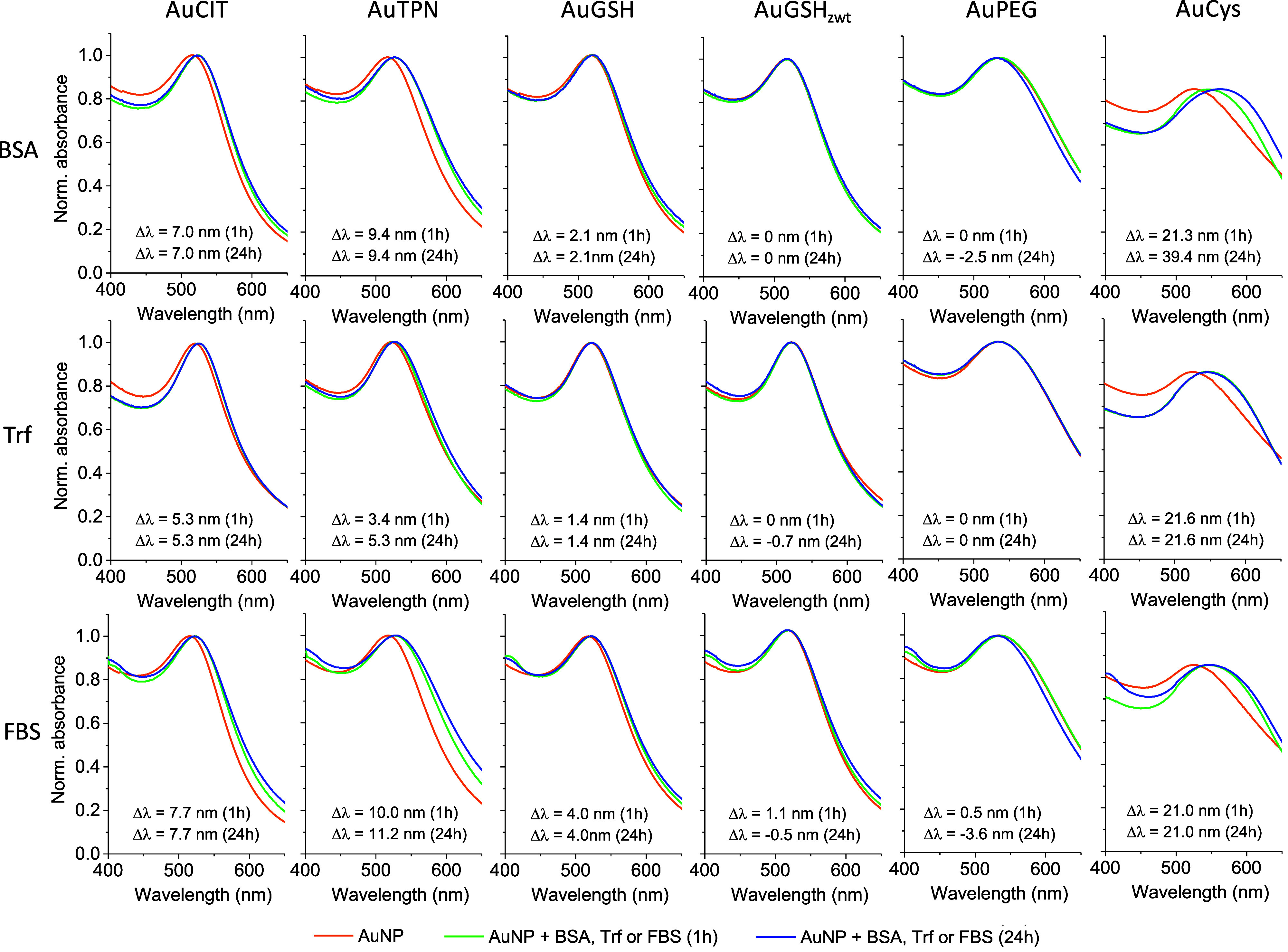
Characterization of hard protein corona formation on AuNPs through UV–vis spectroscopy. AuNPs were incubated in solutions containing BSA (10 mg/mL), transferrin (2 mg/mL), or FBS (30%) for 1 or 24 h and at 37 °C. Subsequently, the AuNPs were centrifuged and washed 3x, followed by redispersion in phosphate buffer. AuNPs dispersed in buffer alone were used as a control. Δλ is the difference in wavelengths at peak intensity for AuNPs in the presence of proteins compared to the buffer control.

Our results indicated the formation of hard protein coronas on AuCIT, AuTPN, AuGSH, and AuCys when exposed to BSA or transferrin. Specifically, the UV–vis analyses demonstrated an obvious red shift of the LSPR band of these AuNPs in the presence of BSA and transferrin (Figure 2), which can be attributed to a change in refractive index near the particle surface owing to protein adsorption. Furthermore, DLS measurements demonstrated a significant increase in the HD of the particles upon exposure to BSA and transferrin (Table 1). Alterations in ZP were also observed after the AuNPs were exposed to the protein solutions. We additionally note that there was no significant difference in the protein corona characteristics of the AuNPs as a function of incubation time (Tables 1 and S1). This agrees with a previous report on the time evolution of protein corona formation on colloidal AuNPs of various sizes.^56^ We therefore conclude that a stable protein corona formed quickly on the surface of AuCIT, AuTPN, AuGSH, and AuCys. In contrast to the aforementioned AuNPs, both AuGSH_zwt_ and AuPEG did not develop a significant protein corona as evidenced by UV–vis analysis and DLS and ZP measurements, even after a 24 h incubation period (Tables 1, S1, and Figure 2).

### Formation of FBS Corona

We extended our investigation of the AuNPs to a more complex biological matrix, namely FBS. The AuNPs were incubated in FBS for 1 and 24 h, followed by centrifugation and washing to remove excess FBS. UV–vis spectroscopy, DLS, and ZP measurements were used to characterize the particles. Combined, our findings revealed that AuCIT, AuTPN, AuGSH, and AuCys were all covered by a hard corona consisting of FBS proteins, while no protein corona was detected on AuGSH_zwt_ and AuPEG (Tables 1, S1, and Figure 2).

**Figure 3:**
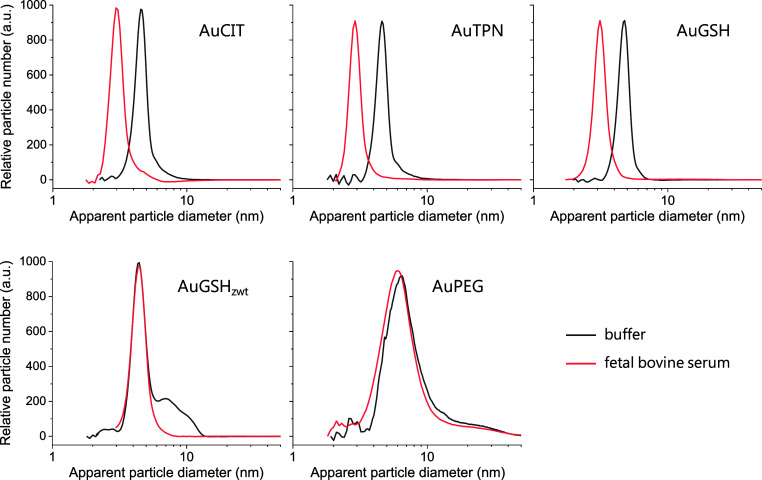
Characterization of protein corona formation on AuNPs via DCS. AuNPs were incubated in buffer or 30% FBS solutions for 1 h at 37 °C and analyzed under in situ conditions using DCS. Traces are the average of three replicates.

Centrifugation and washing to eliminate excess FBS result in the removal of weakly bound proteins from the NP surface. Therefore, the absence of a hard corona on AuGSH_zwt_ and AuPEG does not exclude the possibility of proteins forming weak interactions (soft coronas) with these particles in situ. Investigating these potential weak interactions is crucial, as they can influence the biological responses of NPs.^57−60^ To explore the potential formation of a soft protein corona on AuGSH_zwt_ and AuPEG, we employed differential centrifugal sedimentation (DCS)^61,62^ under in situ conditions. This involved characterizing corona formation without the removal of excess FBS through prior centrifugation and washing steps. The distributions of apparent particle diameters reported by the DCS software are shown in Figure 3 (AuCys was not evaluated because of its colloidal instability). We found smaller apparent particle diameters for AuCIT, AuTPN, and AuGSH in the presence of FBS relative to the control. This confirmed the adsorption of FBS proteins on all three AuNPs. The fact that protein adsorption leads to a reduction, rather than an increase, in apparent diameter is due to an oversimplification embedded in the calculations, which do not take into account the density differences between the corona-coated and pristine AuNPs.^61^ On the other hand, DCS did not reveal obvious changes in apparent particle diameter for AuGSH_zwt_ and AuPEG, thus confirming the “stealth” nature of AuGSH_zwt_ even under these more stringent in situ conditions.

### NP Functionalization

Colloidal AuNPs can be readily functionalized with thiol-containing molecules or cysteine-containing peptides through direct S–Au bond formation, enabling applications like targeted drug delivery. However, the targeting ability of the particles may be partly or totally lost due to steric hindrance from the underlying surface coat. Moreover, the formation of a protein corona could shield the targeting moiety and compromise the targeting ability.^63,64^

**Figure 4:**
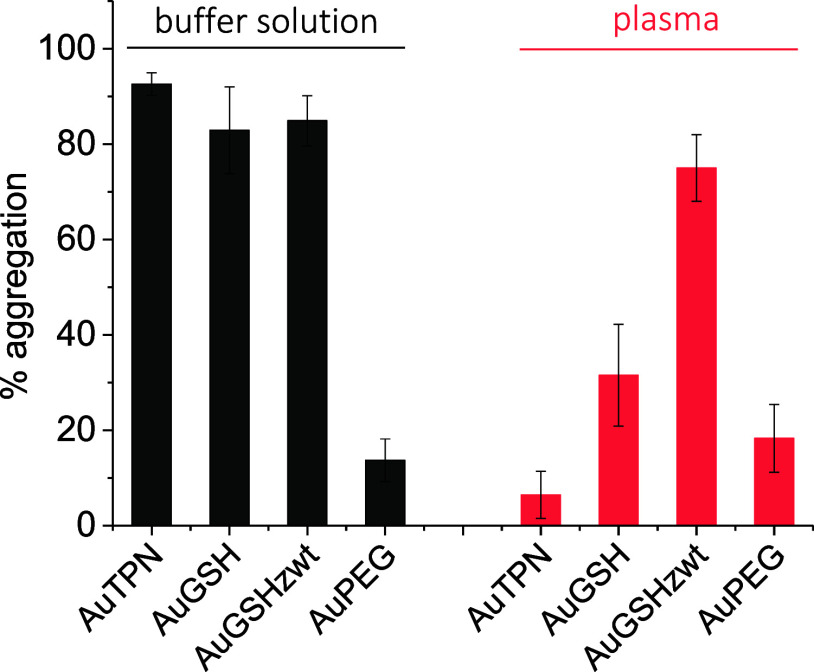
Assessment of the binding of biotin-labeled AuNPs to streptavidin in buffer solution and human plasma. Aggregation of biotinylated AuNPs induced by streptavidin was used as a measure of binding. Percent aggregation was calculated as described in the Experimental Methods.

We investigated whether our various AuNPs could be functionalized and preserve their targeting capacity when dispersed in human plasma. As a model system, the biotinylated peptide Glu–Cys–Gly–Lys–biotin served as the targeting ligand, and streptavidin acted as the corresponding receptor. The various biotinylated AuNPs were dispersed in either a buffer solution or human plasma, followed by the introduction of streptavidin into the system. Aggregation of the AuNPs induced by streptavidin served as an indicator of ligand–receptor interactions (Figure 4).

We observed efficient aggregation/binding of both biotinylated AuTPN and AuGSH to streptavidin in buffer solution but not in human plasma. This indicates that AuTPN and AuGSH were covered by a protein corona in plasma, which shielded the biotin moiety and prevented it from binding to streptavidin. For AuPEG, we observed no binding to streptavidin even in buffer solution, indicating steric hindrance from the bulkier PEG coat on the biotin ligand. It should be noted that stabilizing the AuNPs with a shorter PEG chain, as opposed to the 5 kDa chain used here, could possibly mitigate the steric hindrance effect and enable targeted binding to streptavidin. In the case of AuGSH_zwt_, the results revealed interactions with streptavidin both in buffer and human plasma, suggesting a promising potential for AuGSH_zwt_ in targeting applications.

### Blood Compatibility

Blood compatibility is a critical requirement for NPs that are administered intravenously.^65−69^ Importantly, we note that the absence of a hard protein corona on the NP surface does not in itself ensure blood compatibility. This is because transient NP interactions with proteins are capable of interfering with protein function and biochemical pathways, potentially triggering toxic responses.^49,58−60,70−72^ Moreover, NP interactions with the plasma membrane of blood cells can also perturb their normal functions. Therefore, a comprehensive evaluation of blood compatibility is necessary to assess potential risks associated with NP administration in vivo.^22,73−78^ This assessment should include several complementary assays, including the hemolysis of red blood cells, platelet aggregation, leucocyte activation, and endothelial cell cytotoxicity. Furthermore, any potential perturbations caused by NPs in the coagulation and complement cascade systems must also be evaluated. Here, we outline the experiments concerning the blood compatibility of the various ligand-coated AuNPs, excluding AuCys, which was not further examined.

#### Plasma Coagulation Cascade

Plasma coagulation involves a sequence of enzymatic reactions culminating in the thrombin-mediated cleavage of fibrinogen into insoluble fibrin. AuNPs intended for in vivo applications must be fundamentally “invisible” toward the coagulation system. This is crucial since even minor perturbations to the coagulation cascade can cause life-threatening complications such as bleeding and thrombosis.^65,68^ The latter, in particular, can be triggered by activation of factor XII (FXII) into FXIIa upon contact with a foreign surface. In the related field of biomaterials, unintended blood coagulation through contact activation remains, to this date, a major obstacle to the safe use of implantable medical devices.^79^

**Figure 5:**
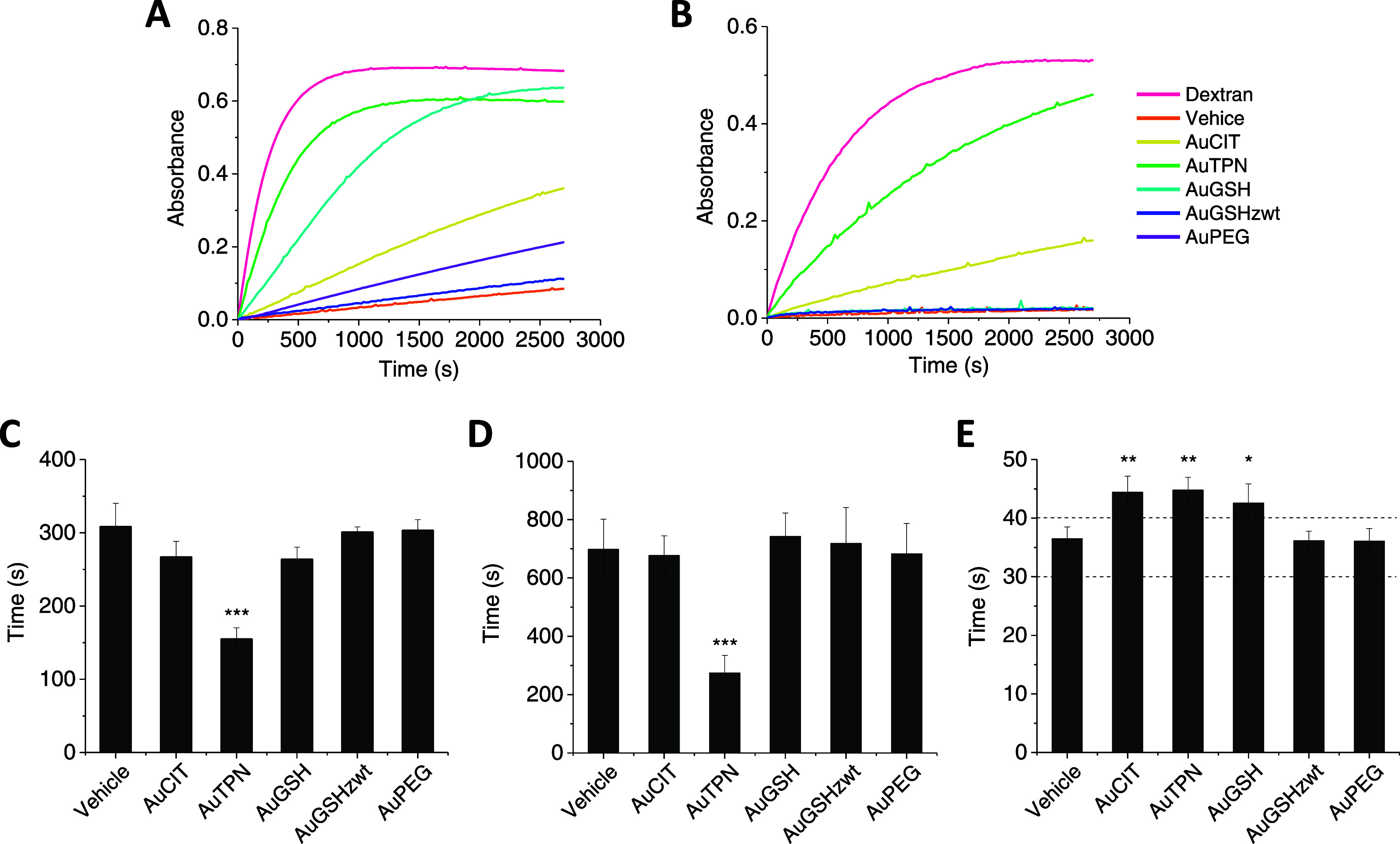
Effects of AuNPs on plasma coagulation. (A) Activation of purified FXII by AuNPs. (B) Activation of FXII/PK in human plasma by AuNPs. In (A,B), FXII or FXII/PK conversion into FXIIa/PKa was assessed by recording amidolytic activities using the substrate S-2302. Dextran sulfate served as a positive control, and buffer alone was used as a vehicle control. (C) AuNP-induced clot formation in human plasma assayed by mechanical coagulometry. (D) AuNP-induced clot formation in human plasma assayed by ROTEM. (E) Impact of AuNPs on the aPTT of human plasma. Dashed lines indicate a typical range of normal aPTT values. Results were analyzed by one-way ANOVA followed by Tukey’s test, with *p < 0.05, **p < 0.01, and ***p < 0.001 with respect to vehicle control.

We initially evaluated the capacity of the various AuNPs to activate the contact system. To achieve this, we incubated the AuNPs with purified zymogen FXII or human plasma and then quantified the resulting amidolytic activity against the chromogenic substrate S-2302 (Figure 5A). We found that AuTPN and AuGSH strongly converted purified FXII into its active form (FXIIa), while FXII activation induced by AuCIT and AuPEG was less effective. In contrast, AuGSH_zwt_ did not show any noticeable influence on FXII activation. When examining the behavior of the AuNPs in plasma, we observed that only AuCIT and AuTPN triggered efficient activation of the contact system (Figure 5B).

Next, we investigated the potential of the AuNPs to induce clot formation in human plasma. The particles were incubated in plasma, and the time to clot formation was assessed using two complementary methods: mechanical coagulometry and rotational thromboelastometry (ROTEM). The findings revealed that only AuTPN visibly reduced the time to clot formation (Figures 5C,D and S4). We also explored the effects of the AuNPs on the activated partial thromboplastin time (aPTT) of human plasma. For this purpose, plasma samples were incubated with the AuNPs and then treated with the aPTT reagent to activate the coagulation cascade. The results showed that AuCIT, AuTPN, and AuGSH caused a slight prolongation of the clotting time, while AuGSH_zwt_ and AuPEG had no effect (Figure 5E).

Taken together, these results suggest that AuCIT, AuTPN, and AuGSH have complex interactions with the coagulation cascade. Specifically, AuCIT activates the contact system, yet it also appears to interfere with downstream reactions, resulting in no observable impact on time to clot formation. Compared to AuCIT, AuTPN exhibited a more potent activation of the contact system, resulting in a shortened clotting time. When the cascade was initiated with a more potent activator (aPTT reagent), AuCIT, AuTPN, and AuGSH slightly prolonged the time to clot formation, implying that they interfere with downstream reactions beyond the point of contact activation. Most importantly, our findings highlight the distinctive “stealth” properties of AuGSH_zwt_ regarding its interaction with the coagulation system. Specifically, AuGSH_zwt_ did not activate the contact system and did not impact the time to clot formation. The observed lack of activation of purified FXII by AuGSH_zwt_ was particularly impressive, suggesting that the GSH_zwt_ layer is highly effective in preventing even short-lived interactions of proteins with the particle surface.

#### Complement System

The complement system serves as the initial defense against invading pathogens by promoting rapid pathogen lysis and opsonization. Furthermore, it helps mount the more specific (but slower) adaptive immune response. Complement proteins exist as inactive precursors that become activated upon encountering a foreign surface. In each of the three major complement-activation pathways, the third complement protein, C3, undergoes cleavage, producing C3b and the smaller fragment C3a.^80^

**Figure 6:**
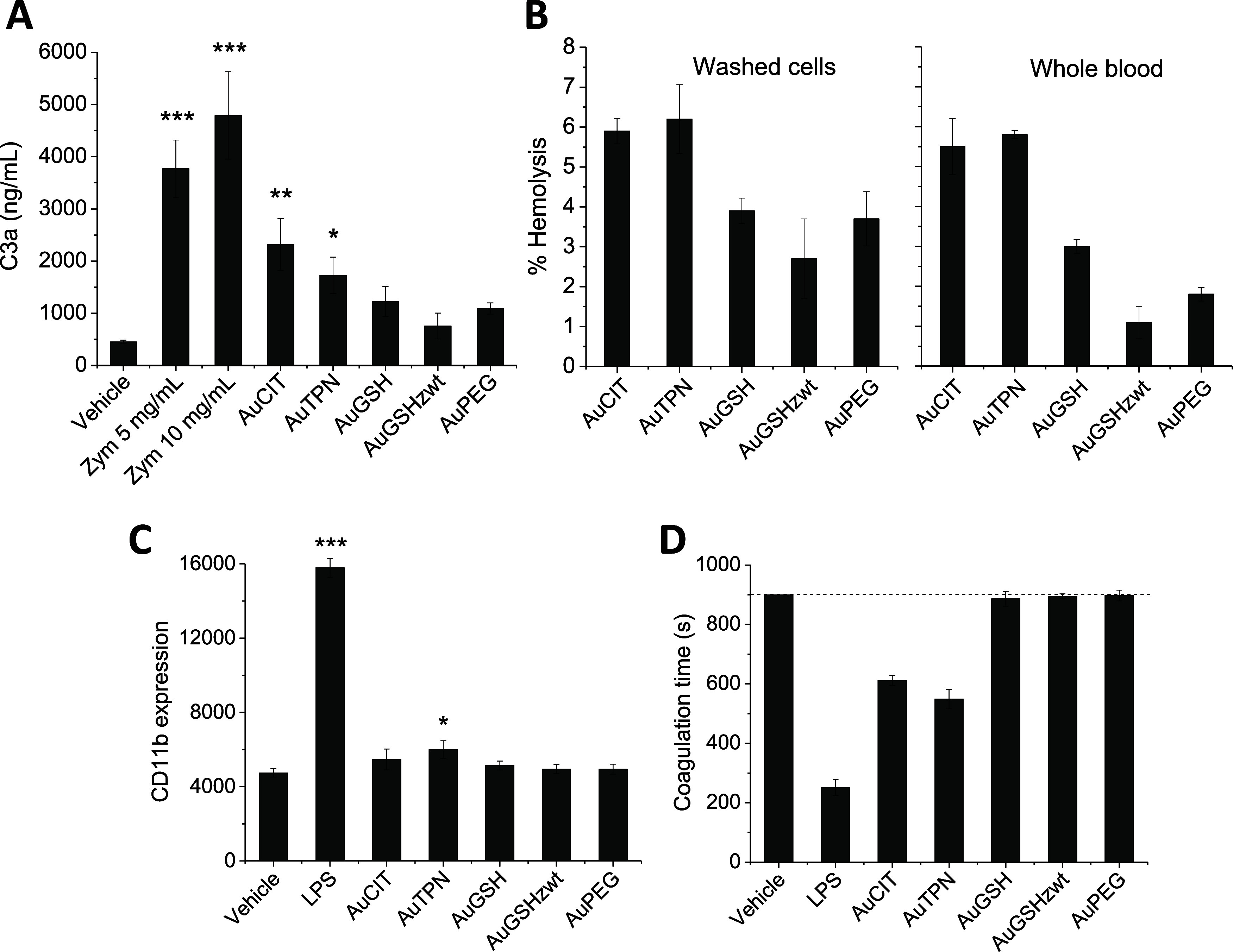
Effects of AuNPs on complement activation, hemolysis, and leukocyte activation. (A) Impact of AuNPs on complement activation (C3a) in human plasma assayed by ELISA. Zymosan (Zym) was used as a positive control. (B) Percent hemolysis of washed erythrocytes or erythrocytes in whole blood treated with AuNPs. (C) Impact of AuNPs on CD11b expression in granulocytes. Blood cells were exposed to AuNPs, stained with anti-CD11b-PE, and analyzed by flow cytometry. (D) Impact of AuNPs on leucocyte PCA. PBMCs were treated with AuNPs and then used to induce coagulation in plasma. Coagulation time was measured using a coagulometer. LPS was used as a positive control. Dashed line indicates the threshold coagulation time under which the treated samples are considered pro-coagulant. Results were analyzed by one-way ANOVA followed by Tukey’s test, with *p < 0.05, **p < 0.01, and ***p < 0.001 with respect to vehicle control.

Unintended activation of the complement cascade by NPs can result in various undesirable outcomes, including the immune clearance of NPs, hypersensitivity reactions, and activation of immune cells.^81,82^ We evaluated complement activation by our ligand-coated AuNPs in human plasma using an enzyme-linked immunosorbent assay (ELISA) kit for the quantitation of C3a. Our results indicated that AuCIT and AuTPN activated the complement cascade, whereas the other AuNPs demonstrated no significant activation compared to untreated plasma (Figure 6A).

#### Hemolysis

Erythrocytes occupy a large volume fraction of blood; hence they are inevitably exposed to administered NPs. Perturbation of erythrocyte membrane integrity through interactions with NPs can lead to hemolysis, resulting in the leakage of hemoglobin into the bloodstream—a potentially life-threatening condition.^66,83^ To probe the occurrence of NP-induced hemolysis, erythrocytes were exposed to AuNPs for 4 h, after which percent hemolysis was calculated as described in the section Experimental Methods. The findings indicated that AuCIT and AuTPN induced considerable hemolysis levels exceeding 5%, while AuGSH_zwt_ exhibited the least hemolytic activity among all particles (Figure 6B).

#### Platelet Aggregation

Platelets play a pivotal role in primary hemostasis and blood clotting. Unintended platelet activation resulting from interactions with NPs may trigger acute thrombotic events such as ischemic stroke and myocardial infarction.^66,83^ To probe the impact of the AuNPs on platelet aggregation, we employed light transmission aggregometry (Figure S5). These experiments involved samples of both platelet-rich plasma and a suspension of washed platelets. We observed that none of the AuNPs initiated platelet aggregation independently under the conditions of the experiments. Furthermore, none of the AuNPs hindered platelet aggregation when this was stimulated by the agonists, arachidonic acid and thrombin.

#### Leukocyte Activation

Leucocytes are categorized into monocytes, granulocytes, and lymphocytes, playing pivotal roles in inflammation, immunity, and hemostasis.^66^ The activation of leucocytes, particularly monocytes and granulocytes, can be identified by changes in the expression levels of membrane proteins such as CD11b.^77^ This protein, which belongs to the integrin family, plays a fundamental role in the phagocytosis of opsonized invaders and can serve as a practical inflammatory marker. The activation of leucocytes can also increase the expression of the pro-coagulant activity (PCA) complex on the cell surface, which can trigger the coagulation cascade and contribute to thrombogenicity.^84^

To assess leukocyte activation, whole blood was exposed to AuNPs, and the expression of CD11b was evaluated through antibody labeling and flow cytometry. Bacterial lipopolysaccharide (LPS) and buffer served as positive and negative controls, respectively. The findings revealed that AuTPN increased CD11b expression levels in granulocytes beyond the negative control, while the other AuNPs had no effect (Figure 6C). No observable impact on CD11b expression levels in monocytes was noted with any of the AuNPs (not shown). To investigate whether the AuNPs could activate the leukocyte PCA, peripheral blood mononuclear cells (PBMCs) were exposed to AuNPs, and PCA was measured in a coagulometer by determining the time for plasma clot formation induced by the treated cells. The results indicated that AuCIT and AuTPN activated leucocyte PCA, evidenced by a significant reduction in plasma coagulation time, while the other particles showed no effect (Figure 6D).

#### Cytotoxicity

The presence of AuNPs in blood circulation raises concerns about potential adverse effects on the endothelial cells lining blood vessels. Therefore, evaluating the cytotoxicity of AuNPs to endothelial cells is a crucial aspect of blood safety assessment. We employed human umbilical vein endothelial cells (HUVECs) to evaluate the cell toxicity response upon contact with the various ligand-coated AuNPs for 24 h. We assessed cell metabolic activity through the MTT calorimetric assay, cell apoptosis through the Annexin V staining method, and cell oxidative stress through the CellROX reagent. We observed that AuCIT and AuTPN induced increased cell oxidative stress compared to the control. However, the remaining AuNPs did not induce any detectable toxicity in HUVECs under the experimental conditions (Figure S6).

## Conclusions

**Figure 7:**
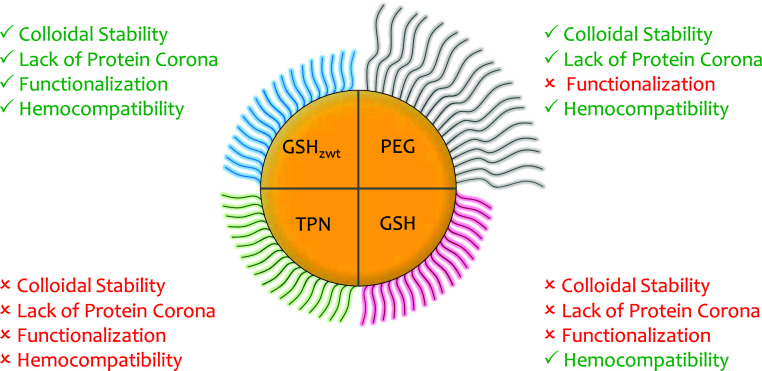
Summary of main results. Evaluation of colloidal stability, resistance to protein corona formation, functionalization potential, and hemocompatibility for various 5 nm sized ligand-coated AuNPs. AuPEG had an HD ∼ 23 nm, while the other particles had HDs ∼ 6.5 nm. Only AuGSH_zwt_ demonstrated optimal performance across all assessments.

We prepared a variety of ligand-coated colloidal AuNPs, introducing novel GSH_zwt_-coated particles, and assessed their behavior in biological media along with their impact on crucial blood compatibility parameters. Figure 7 presents a summary of the main findings. We observed that anionic AuTPN and AuGSH were colloidally unstable under various incubation conditions in protein-free solutions. Moreover, these particles readily acquired a protein corona when exposed to protein solutions. It is interesting to note that the GSH ligand, although widely employed as a surface coating for stable and protein-resistant ultra small AuNPs (<2 nm), was unsuitable as a surface ligand for producing “stealth” colloidal AuNPs. In hemocompatibility tests, it became apparent that the protein coronas formed around AuTPN and AuGSH exhibited distinct characteristics since AuTPN demonstrated poor blood compatibility while AuGSH showed good compatibility across the tested parameters. We also found that despite being coated with a zwitterionic ligand, AuCys displayed poor colloidal stability. AuPEG demonstrated stability, resistance to protein adsorption, and biocompatibility. However, PEG conjugation has well-known drawbacks, including an increase in the HD of particles and the potential to elicit an immunogenic response. In our study, biotin-functionalized AuPEG could not bind to streptavidin receptors owing to steric hindrance effects arising from the PEG surface coating. Finally, AuGSH_zwt_ exhibited excellent stability and resistance to protein adsorption. The particles could also be functionalized with biotin and interact with streptavidin receptors in human plasma. Additionally, they demonstrated exceptional blood compatibility and noncytotoxicity in various in vitro tests. Further in vitro and in vivo studies will be required to establish the safety profile of AuGSH_zwt_. Taken together, we propose that GSH_zwt_—as a commercially available, readily accessible, and biocompatible zwitterionic derivative of GSH—holds promise for the surface passivation of engineered NPs intended for biomedical applications.

## Experimental Methods

### Materials and Reagents

Citrate-stabilized colloidal AuNPs with a diameter of 5 nm were procured from Sigma-Aldrich. The small-molecule ligands glutathione, tiopronin, and cysteine were obtained from Sigma-Aldrich, glutathione monoethyl ester was from Bachem, and thiol mPEG was from Nanocs. Glu–Cys–Gly–Lys–biotin was acquired from LifeTein. Purified FXII and thrombin were acquired from Innovative Research. The chromogenic substrate S-2302 was obtained from Chromogenix. The aPTT reagent was supplied by Siemens Healthcare Diagnostics. Arachidonic acid was obtained from Sigma-Aldrich. Human C3a ELISA kit and anti-CD11b-PE were acquired from Thermo Fisher Scientific/Invitrogen. FACS lysing solution was from BD Biosciences. *Escherichia coli* LPS was obtained from Sigma-Aldrich. Citrated whole blood was collected from healthy donors after approval by the institution’s Research Ethics Committee. The blood samples were subjected to standard centrifugation protocols to obtain platelet-rich and platelet-poor plasma, as well as washed red blood cells and PBMCs. The following buffer solutions were prepared before each experiment following standard protocols: PB (10 mM phosphate, pH 7.4), PBS (20 mM phosphate, 150 mM NaCl, 0.01% tween 20, pH 7.4), Tris–HCl (100 mM, 150 mM NaCl, 0.01% tween 20, pH 7.4), HEPES (100 mM, 150 mM NaCl, 0.01% tween 20, pH 7.4), and HEPES–Tyrode (5 mM HEPES, 137 mM NaCl, 2.9 mM KCl, 12 mM Na_2_HPO_4_, 5 mM C_6_H_12_O_6_, 1 mM CaCl_2_, 1 mM MgCl_2_, pH 7.4).

### Preparation and Characterization of AuNPs

To prepare the various ligand-exchanged AuNPs, AuCIT (20 nM) was treated with TPN, GSH, GSH_zwt_, Cys, or PEG (5 μM) for 2 h at room temperature. Subsequently, the particles were purified through three cycles of centrifugation and washing. Finally, they were redispersed in PB buffer and stored at 4 °C at a concentration of 90 nM for further use. The concentration of 90 nM corresponds to the original concentration provided by the manufacturer, where the particles exhibit an optical density of 1 at 520 nm. The ligand-exchanged AuNPs were characterized according to hydrodynamic size and surface charge through DLS and ZP measurements employing a Malvern Zetasizer Nano ZS instrument. Additionally, the AuNPs were characterized through UV–vis spectroscopy using a Shimadzu UV–1800 spectrophotometer.

### Characterization of Protein Corona through DLS, ZP, and UV–Vis Spectroscopy

The AuNPs (20 nM) were incubated in solutions containing BSA (10 mg/mL), transferrin (2 mg/mL), or FBS (30%) for either 1 or 24 h at 37 °C. Subsequently, the AuNPs were centrifuged and washed 3×, followed by redispersion in PB buffer. The AuNPs with adsorbed protein coronas were characterized using DLS, ZP measurements, and UV–vis spectroscopy.

### DCS of AuNPs

The AuNPs (20 nM) were incubated in PBS or 30% FBS solutions for 1 h at 37 °C. Then, 100 μL of the NP suspensions were injected into the circular sample holder of the DCS equipment (Disc Centrifuge, Mod. DC24000UHR—CPS Instruments Inc.). The disc centrifuge velocity set up was 24 000 rpm. Before sample injection, the centrifugal disc was filled with a sucrose aqueous solution gradient density (density range of 1–8%), and the CPS equipment was calibrated with polystyrene NPs standard sample (mean diameter of 540 nm). Characterization of the AuNPs was performed in triplicate as described above.

### Functionalization of AuNPs with Biotin

The AuNPs were functionalized with the biotinylated peptide Glu–Cys–Cly–Lys–biotin, maintaining a 1:20 ratio of peptide/ligand during ligand exchange of AuCIT. Specifically, AuCIT (20 nM) was treated with TPN, GSH, GSH_zwt_, or PEG (5 μM) in the presence of Glu–Cys–Cly–Lys–biotin (0.25 μM) for 2 h at 25 °C. The remaining ligand exchange procedure was identical as described above. To assess binding of the as-prepared biotin-functionalized AuNPs to streptavidin receptors, the particles (100 nM) were dispersed in either buffer solution or human plasma for 1 h and subsequently mixed with streptavidin (1 μM) for an additional 1 h period. Subsequently, the solution was centrifuged (500*g*, 10 min) to precipitate any formed aggregates, and absorbance readings were obtained of the supernatant at 510 nm (Abs_biotin_). A similar procedure was performed with the non-functionalized AuNPs, yielding absorbance readings in the absence of any aggregation (Abs_ctr_). Percent aggregation, used as a measure of binding, was calculated according to the formula: % aggregation = 100 × (Abs_ctr_ – Abs_biotin_)/Abs_ctr_.

### FXII Activation by AuNPs

To probe FXII activation by AuNPs, purified FXII (100 nM) was incubated with the AuNPs (20 nM) in Tris–HCl buffer for 30 min at 37 °C. Subsequently, amidolytic activities were recorded using the chromogenic substrate S-2302 (300 μM) in continuous mode, using a Spectramax plate reader from Molecular Devices. Dextran sulfate (5 nM) served as a positive control, and buffer alone was the vehicle control. To evaluate the AuNP-induced activation of FXII/PK in human plasma, citrated plasma samples (30% in HEPES buffer) were incubated with the AuNPs and analyzed in a similar manner as described above. Of note, the substrate S-2302 is cleavable by both FXIIa and PKa.

### Time to Clot Formation

AuNP-induced clotting of human plasma was assessed by two complementary techniques, mechanical coagulometry and ROTEM. In both cases, citrated human plasma (30% in HEPES buffer) was incubated with or without the AuNPs (20 nM) for 30 min at 37 °C. After recalcification using CaCl_2_ (6.25 mM), time to clot formation was recorded using a Dade Bhering BFT II coagulometer or a computerized ROTEM four-channel system (Pentapharm). For ROTEM analysis, the time taken for the clot to reach an amplitude of 2 mm served as the measure of clotting time.

### aPTT Assay

Citrated human plasma was incubated with or without the AuNPs (20 nM) for 30 min at 37 °C. Next, samples were treated with the aPTT reagent plus CaCl_2_ (6.25 mM) to initiate clotting. The final volume percentages of plasma and the aPTT reagent were both 25% (v/v). Time to clot formation was recorded using a Dade Bhering BFT II Analyzer.

### Complement Activation

Citrated human plasma (30% in HEPES buffer) was incubated with or without the AuNPs (20 nM) for 30 min at 37 °C. Complement activation through the alternative pathway was evaluated by measuring the generation of complement C3a using ELISA, following the manufacturer’s protocol. Samples containing zymosan (5 and 10 mg/mL) served as positive controls, and buffer alone was the vehicle control.

### Platelet Aggregation

Platelet aggregation experiments utilized samples of platelet-rich plasma and washed platelets. Cell concentrations were adjusted to 2.5 × 10^8^ platelets/mL with HEPES–Tyrode buffer using a Sysmex KX–21N hematological cell counter. Samples were treated with the AuNPs (20 nM) for 30 min at 37 °C, followed by the addition of arachidonic acid (0.5 mM) or human α-thrombin (5 nM) agonists (the latter was added to samples of washed platelets only). Platelet aggregation was analyzed at 37 °C using a Chrono–Log 490 aggregometer (Chrono–Log Corporation). The degree of platelet aggregation was expressed as the percent change in light transmittance from platelet-rich plasma (0% light transmission) to platelet-poor plasma or buffer (100% light transmission). Control measurements were performed at the beginning and end of each experiment to confirm platelet viability.

### Hemolysis

Whole blood samples were diluted in PBS with a volume ratio of 1:50 and incubated with the AuNPs (20 nM) for 4 h at 37 °C in an orbital shaker. Negative controls lacked AuNPs, while positive controls contained 1% Triton X-100. Following incubation, blood samples were centrifuged at 1500 g for 5 min, and supernatants were analyzed in a microplate reader with absorbance readings at 540 nm. A similar procedure was performed using washed red blood cells suspended in PBS. Percent hemolysis was calculated using the formula: % hemolysis = 100 × [(OD_sample_ – OD_neg_)/(OD_pos_ – OD_neg_)].

### Leukocyte CD11b Expression

For evaluation of granulocyte and monocyte activation, blood samples (1.0 mL) were diluted twice in PBS and incubated with the AuNPs (20 nM) for 4 h at 37 °C under mild agitation. LPS (2 μg/mL) and PBS served as positive and negative controls, respectively. Subsequently, cells were stained with anti-CD11b–PE (dilution 1:400) for 1 h at room temperature. Erythrocytes were lysed by treating the blood samples with a hypotonic FACS lysing solution for 5 min. Cells were washed twice with PBS, resuspended to a final volume of 300 μL, and analyzed by flow cytometry, with granulocytes and monocytes identified based on their forward- and side-scatter signals.

### Leukocyte PCA

The assessment of leukocyte PCA followed standard protocols.^65,76^ Briefly, PMBCs were isolated from freshly drawn human blood using gradient separation with Ficoll–Paque Plus (Cytiva). Subsequently, the PBMCs (3 × 10^6^ cells mL^–1^) were washed and redispersed in RPMI medium supplemented with 10% FBS. The cells were then incubated with the AuNPs (20 nM) for 24 h at 37 °C in a 5% CO_2_ atmosphere. LPS (2 μg/mL) and PBS were used as positive and negative controls, respectively. After the incubation period, cells were washed and resuspended in HEPES buffer supplemented with 6.6 mM CaCl_2_. The cells were then used to induce coagulation in human plasma. For this purpose, the cells were mixed with an equal volume of plasma, and the time to clot formation was measured using an automatic coagulometer.

### Cytotoxicity

HUVECs were cultured in DMEM/F12 Glutamax supplemented with 10% FBS and 1% penicillin/streptomycin at 37 °C in a humidified 5% CO_2_ atmosphere. The effect of the AuNPs on cell viability was evaluated using the MTT calorimetric assay and the Annexin V/propidium iodide (PI) staining method while the influence of the particles on cell oxidative stress was assessed using the CellROX reagent. Accordingly, cells were seeded in 96-well plates (MTT assay) or 24-well plates (Annexin V, CellROX) the day prior to the experiments. Then, the cell medium was replaced with fresh medium containing 10% FBS and the AuNPs (20 nM), and cells were exposed to the particles for a 24 h incubation period. Samples lacking AuNPs were employed as controls. The assessment of cell viability using the MTT method was performed according to standard protocols. Cell viability with Annexin V/PI was determined via flow cytometry, where events were gated, and cells outside the control gate (e.g., double-negative) were categorized as either apoptotic (Annexin V positive) or necrotic (PI positive), with results presented as a percentage of these gates. Cell oxidative stress was evaluated using flow cytometry with the CellROX kit, where positive cells were pretreated with terc-butyl hydroperoxide (200 μM) for 2 h before collection, and results were recorded as the median fluorescence intensity of the CellROX reagent.

## Supporting Information Available

DLS and ZP characterization of protein corona formation on AuNPs; UV–vis spectroscopy characterization of AuNPs as a function of pH or dispersed in DMEM; ROTEM data to assess time to clot formation; evaluation of platelet aggregation in the presence of AuNPs; and cytotoxicity assessment in HUVECs (PDF)
