# Supplementary material for: Stealth and Biocompatible Gold Nanoparticles through Surface Coating with a Zwitterionic Derivative of Glutathione
Source: Langmuir. 2024 May 29;40(23):12167–78. doi: 10.1021/acs.langmuir.4c01123 (PMC11171461; doi:10.1021/acs.langmuir.4c01123)
Supplement: Supplementary file 1 — la4c01123_si_001.pdf [file la4c01123_si_001.pdf]

Stealth and Biocompatible Gold Nanoparticles Through Surface Coating with a Zwitterionic Derivative of Glutathione

Vinicius S. Guido,[1] Paulo H. Olivieri Jr,[1] Milena L. Brito,[2] Benedito C. Prezoto,[3] Diego S.T. Martinez,[2] Maria Luiza V. Oliva,[1] Alioscka A. Sousa[1,*]

[1] Department of Biochemistry, Federal University of São Paulo, São Paulo, SP 04044-020, Brazil

[2] Brazilian Nanotechnology National Laboratory (LNNano), Brazilian Center for Research in Energy and Materials (CNPEM), Campinas, SP 13083-100, Brazil

[3] Laboratory of Pharmacology, The Butantan Institute, São Paulo, SP 05503-900, Brazil

* alioscka.sousa@unifesp.br

Supplementary Table S1. Characterization of protein corona formation on AuNPs through DLS and ZP measurements. AuNPs were incubated in solutions containing BSA (10 mg/mL), transferrin (2mg/mL), or FBS (30%) for 24h at 37 °C. Subsequently, the AuNPs were centrifuged and washed 3x, followed by redispersion in phosphate buffer solution. AuNPs dispersed in buffer alone were used as a control.

AuNP	Buffer		BSA corona		Transferrin corona		FBS corona	
	Diameter (nm)	ZP (mV)	Diameter (nm)	ZP (mV)	Diameter (nm)	ZP (mV)	Diameter (nm)	ZP (mV)
AuCIT	5.1 ± 0.9	-33.9 ± 5.8	23.8 ± 2.4	-21.8 ± 2.4	16.6 ± 1.4	-28.1 ± 2.9	24.4 ± 3.3	-28.5 ± 1.8
AuTPN	6.4 ± 1.2	-23.9 ± 4.9	23.9 ± 4.9	- 27.9 ± 6	15.7 ± 1.3	-32.0 ± 3.5	28.4 ± 4.2	-31.4 ± 2.6
AuGSH	6.4 ± 1.2	-20.8 ± 5.6	24.0 ± 1.8	-27.0 ± 1.8	12.6 ± 0.7	-22.8 ± 3.1	26.4 ± 3.9	-29.3 ± 1.9
$AuGSH_{zwt}$	6.6 ± 0.9	0.4 ± 2.5	6.7 ± 1.0	3.4 ± 3.8	6.9 ± 2.0	-2.1 ± 2.5	6.5 ± 1.0	0.57 ± 4.0
AuPEG	22.6 ± 4.7	-24.2 ± 3.0	23.5 ± 4.3	-23.9 ± 3.8	24.0 ± 6.3	-24.0 ± 3.7	24.8 ± 5.0	-23.8 ± 2.7
AuCys	20.7 ± 2.2	-8.8 ± 1.6	73.0 ± 11	-20.9 ± 2.6	45.3 ± 8.0	-24.4 ± 4.5	40.1 ± 2.9	-20.4 ± 4.4

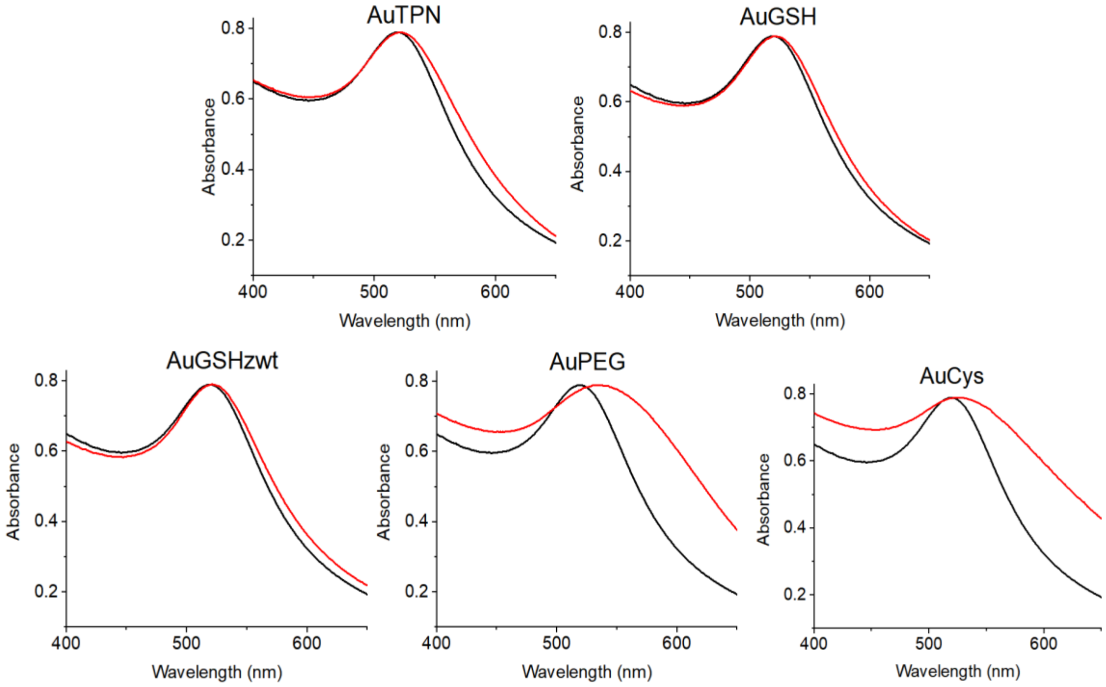

Figure S1. Characterization of AuNPs by UV-visible spectroscopy. Black, AuCIT; red, ligand-exchanged AuNPs.

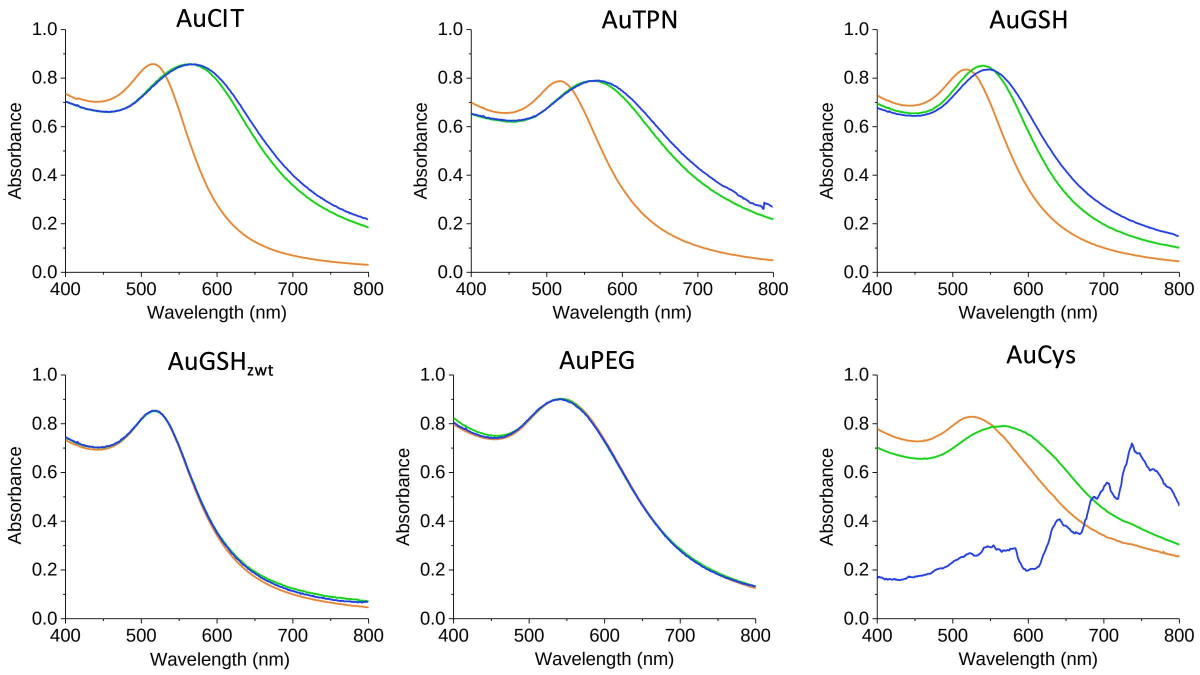

Figure S2. AuNP stability in DMEM cell culture medium. Red, buffer control; green, 1h incubation in DMEM; blue, 24h incubation in DMEM.

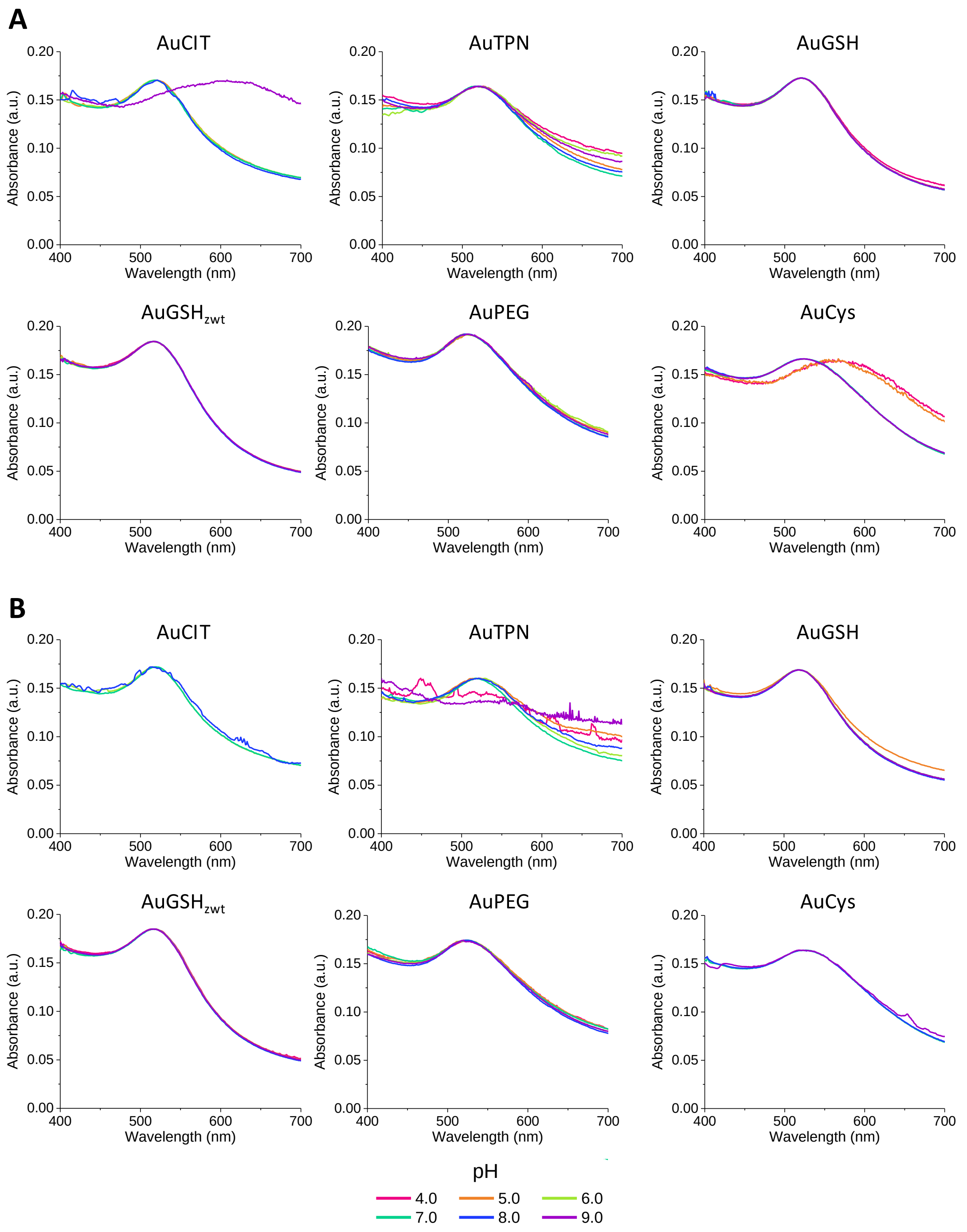

Figure S3. AuNP stability as a function of pH assessed by UV-vis spectroscopy. Solutions were incubated for (A) 1 h and (B) 24 h before measurements. The UV-vis spectra of severely aggregated particles are not shown (AuCIT and AuCys at 24 h).

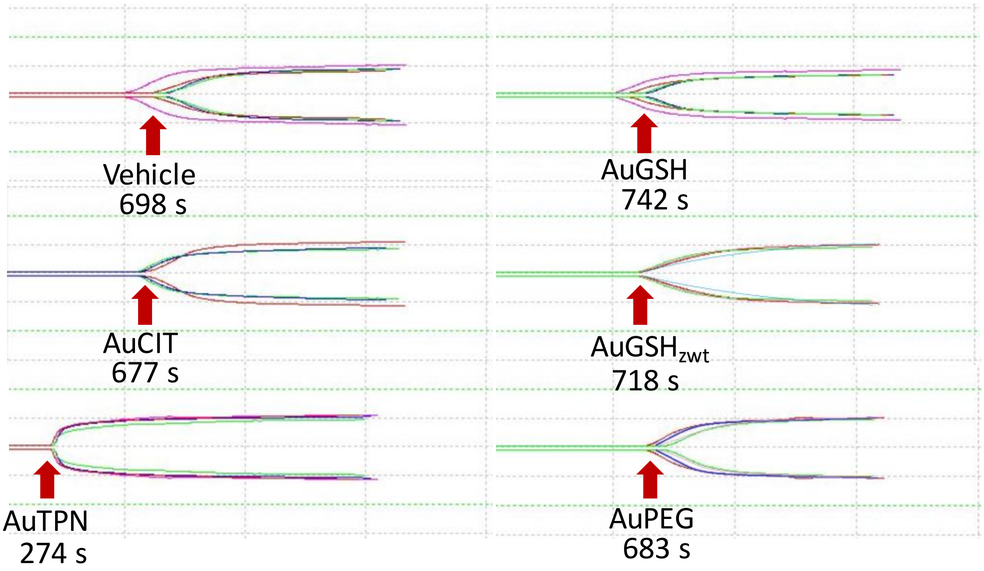

Figure S4. Time to clot formation in human plasma assessed by rotational thromboelastography (ROTEM). Exemplary ROTEM data obtained from samples of human plasma treated with AuNPs (20 nM). Average clotting times are indicated by red arrows. See also Fig. 5D within the main text.

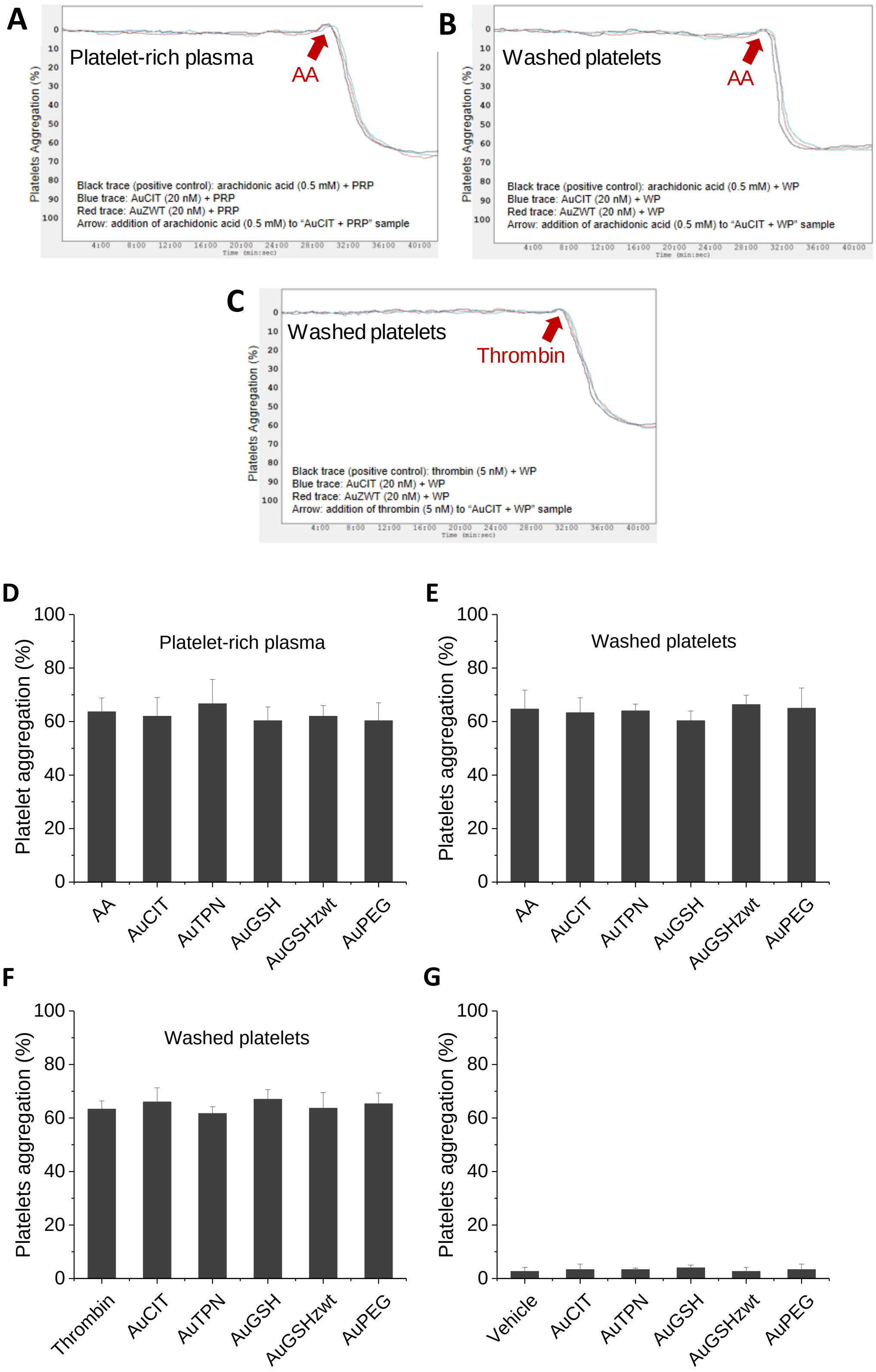

Figure S5. Effects of AuNPs on platelet aggregation. Suspensions of platelet-rich plasma (PRP) or washed platelets (WP) were incubated with AuNPs (20 nM) for 30 min at 37 °C. Subsequently, samples were treated with either arachidonic acid (AA; 0.5 mM) or thrombin (5 nM) to induce platelet aggregation, with thrombin added only to samples of WP. (A-C) Light transmission aggregometry measurements are shown for AuCIT and

$AuGSH_{zwt}$ according to the details in the figure. Arrows denote the point of AA or thrombin addition. Similar outcomes were observed for the other AuNPs. (D-F) Compilation of percent platelet aggregation data demonstrating that none of the AuNPs hindered platelet aggregation when this was stimulated by AA or thrombin. (G) Compilation of percent platelet aggregation data demonstrating that none of the AuNPs initiated platelet aggregation independently.

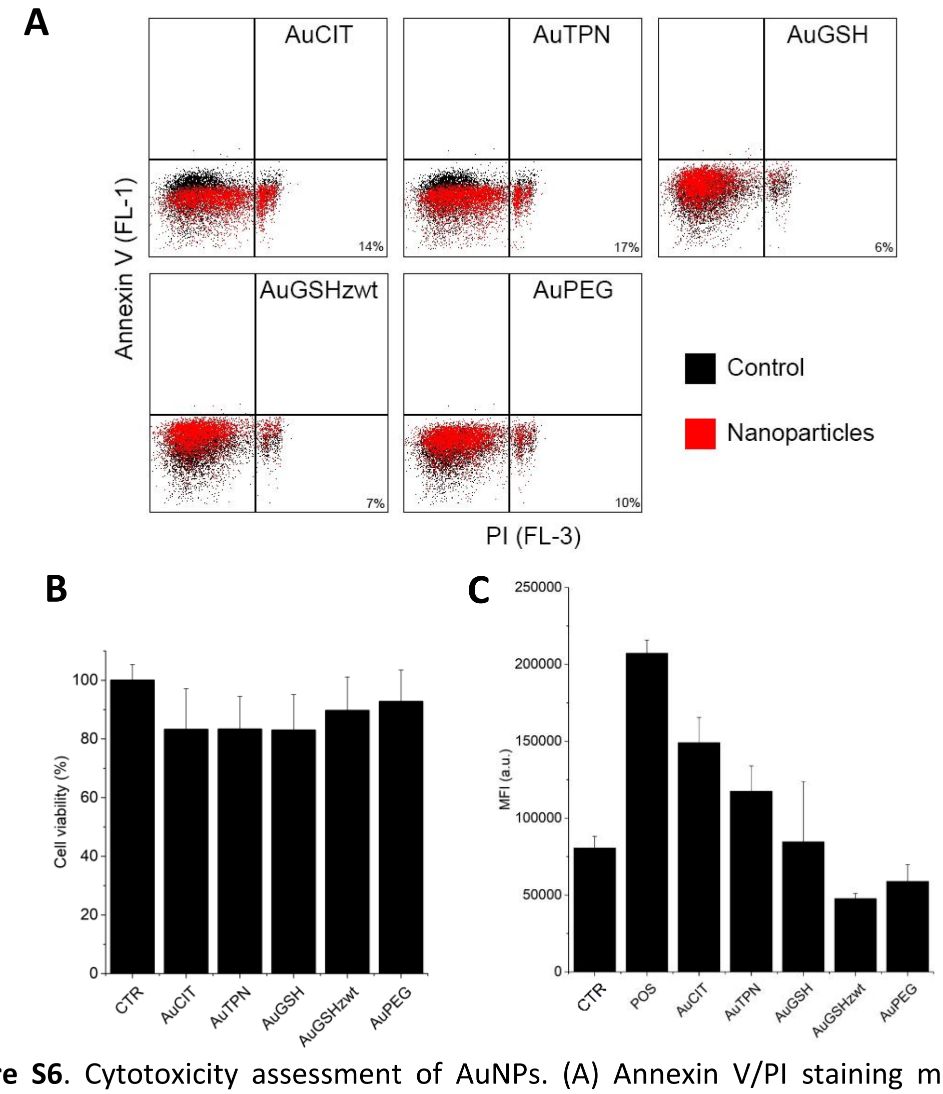

Figure S6. Cytotoxicity assessment of AuNPs. (A) Annexin V/PI staining method for determination of apoptotic and necrotic cells. The percentage of PI positive cells are indicated in each plot; Annexin V positive cells were not observed. (B) MTT calorimetric assay. (C) Cell oxidative stress using the CellROX kit. AuCIT and AuTPN produced increased cell oxidative stress relative to the negative control (CTR). Positive cells (POS) were pre-treated with terc-butyl hydroperoxide. In all assessments, cells were exposed to AuNPs (20 nM) or PBS (CTR) for 24 h in cell culture medium containing 10% FBS.
